# Phenolic compounds of blueberries (*Vaccinium spp)* as a protective strategy against skin cell damage induced by ROS: A review of antioxidant potential and antiproliferative capacity

**DOI:** 10.1016/j.heliyon.2021.e06297

**Published:** 2021-02-17

**Authors:** Daniela A. Maya-Cano, Sandra Arango-Varela, Gloria A. Santa-Gonzalez

**Affiliations:** Biomedical Innovation and Research Group, Faculty of Applied and Exact Sciences, Instituto Tecnológico Metropolitano, Medellín, Colombia

**Keywords:** *Vaccinium ssp.*, Phenolic compounds, Antioxidant activity, Antiproliferative effect, ROS, Skin, Cancer

## Abstract

The skin is a tissue with a high metabolic activity that acts as a protective layer for the internal organs of the body. This tissue is exposed to a variety of damaging agents, including reactive oxygen species (ROS), which can lead to oxidative damage to various macromolecules, disrupting vital cellular processes and increasing mutations. A situation referred to as oxidative stress occurs when a large amount of oxidants exceeds the capacity of the antioxidant defense system. Oxidative stress is considered a contributory factor to the aging process and the pathogenesis of various skin diseases, including cancer. Several current studies seek to identify new natural compounds with properties that mitigate the harmful effects of ROS, thereby acting as blockers or suppressors of the carcinogenesis process. This review briefly presents the relationship between ultraviolet radiation, ROS, and skin damage; and summarizes the in vitro and in vivo experimental evidence of the chemopreventive effect on skin cancer of phenolic compounds from blueberries (*Vaccinium spp*). Although several studies addressed the topic of bioactive compounds and their activities as possible anticancer agents, none have focused on the antioxidative action and antiproliferative effects on skin cancer of phenolic compounds derived from blueberries.

## 1 Introduction

The skin is a tissue with a high metabolic activity that acts as a protective layer for the internal organs. This tissue offers both physical and biochemical protection and is equipped with many defense mechanisms [1]. On the other hand, it is an important target for toxic, physical and chemical agents that can alter its structure and function [2]. These harmful agents include reactive oxygen species (ROS), which can cause oxidative damage to proteins, nucleic acids and lipids, disrupting vital cellular processes and increasing mutations. It is known that prolonged exposure to ultraviolet radiation (UV) emitted by the sun generates ROS that are highly toxic and an underlying cause of damaging effects on the skin. A range of studies demonstrated that the stimulation of melanin caused by UV radiation, and the imbalance between ROS and the antioxidant defense system, are important biochemical contributory factors to the risk of developing skin cancer [3].

Skin cancer is among the tumors with the highest incidence, affecting millions of people internationally. Moreover, the number of cases is increasing at an alarming rate. According to WHO data, one in three cancer cases diagnosed is of the skin [4]. The increase in the incidence of this pathology in recent decades is strongly related to the increasing popularity of open-air activities, as well as to recreational sun exposure. The main factors related to the predisposition to developing this pathology appear to be exposure to sun and a history of sunburns, factors that are within the responsibility of each individual [5, 6]. Currently, there is a need to evaluate new chemopreventive agents that reduce the incidence of skin cancer and that are easily accessible to people frequently exposed to UV radiation through sun exposure.

This review aims to summarize the information available on the benefits of phenolic compounds present in berries of the *Vaccinium* genus and on its role in the protective response mechanisms to skin damage caused by ROS. The data available support the idea that various compounds in these berries have antioxidant and antiproliferative capacities that promote skin health and provide protection against skin damage, including the carcinogenesis process.

## 2 Oxidative stress and pathological effects on skin cells

Reactive oxygen species (ROS) are produced continuously as secondary products of certain metabolic pathways, as well as through some specific systems under fine cellular control, such as pathological inflammation processes [7], oxidases, cytokines [8], peroxisomes, xanthine oxidase (XO), NADPH oxidase [9], acetyl CoA oxidase, and cytochromes [10, 11]. Moreover, in various types of cancer, higher production of ROS is detected and related to activation of pro-tumorigenic signaling, increased cellular survival, proliferation, and the induction of DNA damage, leading to genetic instability [12, 13, 14, 15, 16, 17, 18].

Oxidative stress is caused by an imbalance between oxidants and antioxidants in favor of oxidants, leading to interruption in redox signaling and molecular damage [19]. This definition is in accordance with the fact that the biological functions of reactive species that cause oxidative stress include fundamental processes such as the signaling of cell division and controlled cell elimination in the renewal of tissues. In this way, increased levels of oxidants could alter normal physiological signaling and damage the macromolecular machinery [20]. Generally, there is a balance between the generation and degradation of ROS. Cellular control mechanisms such as the permeability of cellular membranes to reactive species and up-regulation of antioxidant and associated enzymes enable that ROS are found in low quantities in cells (<10^−8^M) [21]. However, oxidative stress is caused when there is excessive production of ROS or where the antioxidant cell defense system is insufficient to neutralize them [22]. This results in damage to vital processes such as replication, transcription and translation [23], as well as causing an increase in mutation, cell death, various pathological processes, and aging [24].

Various types of ROS exist that are generated through the partial reduction of the oxygen molecule during metabolic processes. The superoxide anion (O_2_^-^), which is the primary species generated from the mitochondrial respiratory chain, is moderately reactive and can interfere with cellular components, and can also be converted by the mitochondrial superoxide dismutase to hydrogen peroxide (H_2_O_2_) [25]. This in turn can be transformed into the hydroxyl radical (–OH), which is extremely reactive and has a potential effect against cellular components, causing oxidative damage such as lipid peroxidation, oxidative protein modification, and oxidative damage *per se* in DNA [7, 26].

The main physical agents that generate ROS are ultraviolet and ionizing radiation. Exposure to these induces direct radiolysis of atoms and molecules in the cell, mainly of water, leading to the immediate production of –OH and H_2_O_2_. Also, these forms of radiation increase the intracellular level of ROS after several hours of exposure, which indicates that they also stimulate the endogenous production of oxidant species [27, 28].

Human skin, and particularly the epidermis, is continuously and directly exposed to numerous environmental agents that are chemical and physical inductors of oxidative stress. Of these factors, solar ultraviolet radiation is the most harmful environmental factor, causing the production of ROS and the induction of DNA damage that can eventually lead to carcinogenesis [29, 30]. Skin appears to be endowed with a variety of enzymes and small antioxidant molecules that can inhibit oxidative damage. However, the antioxidant capacity of the skin is often insufficient when faced with excessive production of ROS, which favors the induction of oxidative modifications and damage to macromolecular cells. There are several harmful effects associated with this, including three particularly noteworthy reactions that induce cellular lesions:

### 2.1 Lipid peroxidation in the membranes

In the presence of O_2_, free radicals can cause the peroxidation of the lipids of plasmatic membranes and organelles. The oxidative lesions begin when the double bonds of the unsaturated fatty acids of the lipids of the membranes are attacked by ROS, especially by ^−^OH. The interactions between the free radicals and the lipids generate peroxides, which in turn are unstable and reactive and, consequently, the autocatalytic reaction takes place, which can generate extensive lesions of the membrane [31, 32].

### 2.2 Oxidative modification of proteins

ROS can initiate the oxidation of the lateral chains of amino acids, the formation of disulfide bridges, and the oxidation of the protein backbone. Oxidative modification of the proteins can cause lesions to the active sites of the enzymes, altering the shape of the structural proteins and promoting the degradation by proteasomes of the unfolded or badly folded proteins [33].

### 2.3 DNA lesions

The forms of DNA damage caused by ROS include the oxidative modification of bases and sugar phosphates, the formation of adducts, and the induction of breaks in the single-strand (SSB) and double-strand (DSB) [34]. Unless DNA damage is repaired or eliminated, it can become mutagenic or inhibit replication. For this reason, oxidative DNA lesions are linked to cell aging and malignant transformation [35].

### 2.4 Ultraviolet radiation, ROS and cutaneous damage

The atmosphere is increasingly losing its ability to provide an effective filter, due to decreasing levels of ozone. This causes an increase in solar radiation that arrives at the surface of the earth [36]. Around 7% of the energy emitted by the sun to the earth is ultraviolet (UV) radiation. UV radiation is an invisible form of radiant energy that covers the range of wavelengths between 100 and 300 nm. It is grouped into three categories according to wavelength, as follows: ultraviolet C (UVC, 200–290 nm), ultraviolet-B (UVB, 290–320 nm), and ultraviolet- A (UVA, 320–400 nm) [37, 38, 39].

The shorter the wavelength of UV radiation, the greater its biological harmfulness. UVC does not reach the surface of the earth, as it is absorbed by the ozone layer and, therefore, does not cause cutaneous damage. Meanwhile, UVB causes mutations and has immunosuppressive effects that are essential for carcinogenesis. This type of radiation induces DNA damage that causes modifications in the expression of oncogenes and tumor-suppressor genes, which are probably the most important events in the initiation of cutaneous tumors. As well as this direct effect of UVB on DNA, it has many other effects linked to the production of ROS, including hydrogen peroxide and superoxide anions that can induce single strand breaks, modifications in purine bases, lipid peroxidation in membranes, and oxidative modification in proteins [36, 38, 40]. UVA radiation affects the dermic and epidermic chromophores and induces persistent genomic instability in human keratinocytes through an oxidative stress mechanism that can alter DNA, produce breaks and, ultimately, cause mutations. The main oxidative DNA damage induced by ultraviolet radiation is 8-oxoguanine (8-oxoG). UVA triggers the production of ROS (including singlet oxygen, O_2_) and consequently induces oxidation in the guanine base. This modified guanine is frequently miss-pairing with adenine during replication increasing the number of G–C → T–A transversion mutations, that are directly related to susceptibility to developing cutaneous tumors [24, 39, 41].

### 2.5 Skin cancer

Exposure to ultraviolet radiation and the consequent production of ROS is the etiological factor that is frequently implicated in the development of skin cancer. This disease is divided broadly into two groups: non-melanoma skin cancer, which includes basal cell carcinoma and squamous cell carcinoma, and has a worldwide reported incidence of between 1 and 2 million new cases annually; and melanoma, the third most common form of skin cancer, with approximately 300,000 cases reported annually worldwide [36]. Skin tumors are the most common type of human malignancy and, although most cases are non-melanoma tumors, the less common malignant melanoma is very aggressive and has the highest mortality rate of skin cancers [40].

The development of cancer is a complex process that includes the stages of initiation, promotion and progress, each step mediated by different cellular, biochemical and molecular changes. For skin cancer, is reported that ROS are involved in all three stages of carcinogenesis [42, 43]. In the initiation stage, the extensive oxidative DNA damage in skin cells can cause mutations, including that initiated by the 8-oxoguanina (8-oxoG) lesion which, if not repaired efficiently, can induce GC-TA transversion. This occasional mutation is commonly observed in the tumor suppressor gene p53, which is frequently associated with the pathogenesis of skin cancer [44, 45]. The stages of promotion and progression, which entail the proliferation and malignant transformation of cells, are also promoted by ROS through induction of the overexpression of the Ras and c-Myc oncogenes, which in turn induce overproduction of oxygen free radicals [46, 47]. The potency of ROS in the progression of skin cancer is also related to its capacity to increase the epidermic peroxidation levels and to cause double-strand DNA breaks that accelerate the progress towards malignancy [43].

As ROS are implicated in all the stages of skin cancer carcinogenesis, improvement of the antioxidant capacity of this tissue could protect the cells from the harmful effects of oxidative stress. For this reason, chemopreventive alternatives involving the use of exogenously-administered antioxidants are considered.

## 3 Chemoprevention

Well-known treatments for skin cancer include chemotherapy, immunotherapy and targeted therapy. However, due to the high cost and excessive toxicity of these treatments, various studies have sought to implement new therapeutic strategies, including chemoprevention [48].

Chemoprevention is defined as the use of natural, synthetic or biological substances to reverse, suppress or prevent the initial phases of carcinogenesis, or the progression of premalignant cells to invasive disease [49, 50, 51]. Compounds that inhibit the initiation of cancer are traditionally known as “blocking agents”. These can act by preventing interaction between carcinogenic chemicals or endogenous free radicals and DNA, thereby reducing the level of damage and resulting mutations that contribute not only to the onset of cancer but also to genomic instability and general malignant transformation. This protection can be achieved through a reduction in cellular uptake and the metabolic activation of carcinogens and/or the detoxification and elimination of ROS [52, 53, 54].

The use of nutritional phytochemicals able to block, delay or reverse the process of carcinogenesis has been promoted as an important chemopreventive strategy. Many of these compounds are present in fruits, vegetables, whole grains, nuts, seeds, and legumes and are classified as blockers of the initiation phase or suppressors of the promotion and progression stages of cancer [51]. Phytochemicals include terpenes, phenolic acids and thiols, lignans, and flavonoids. The last of these groups is the most relevant and includes anthocyanins. These compounds are, in general terms, those that provide color and flavor to fruits and vegetables, as well as protecting plants from the various types of stress to which they can be subjected [55].

### 3.1 Berries as a source of chemopreventive compounds

**Figure 1:**
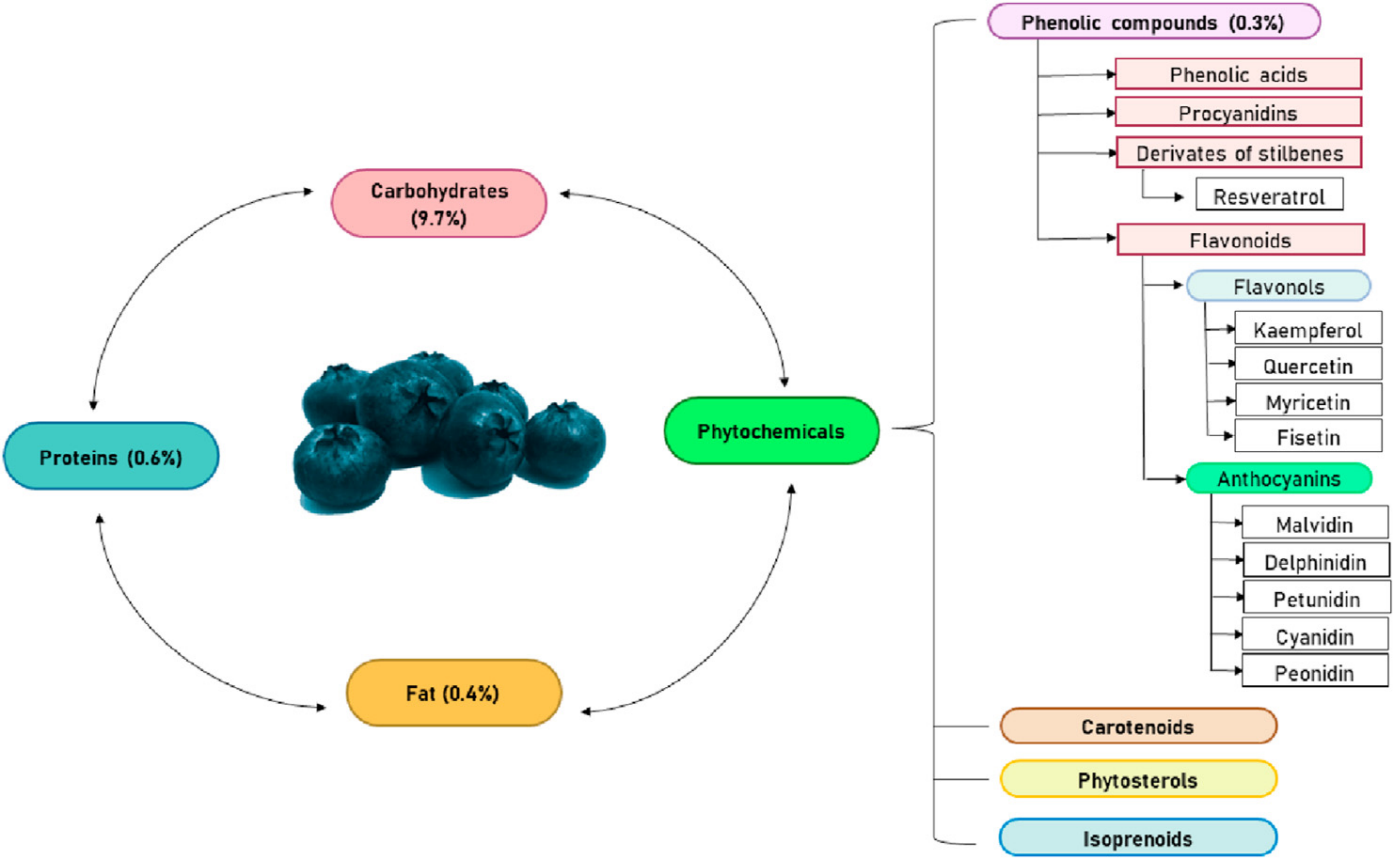
Typical chemical composition of blueberries.

Berries *(Vaccinium spp)* are a class of fruits consumed by humans. These comprise approximately 450 species, which include blueberries, blackberries and cranberries. Fresh blueberries contain carbohydrates, proteins, fat, as well as a high content of water (approximately 84%), and are a significant source of phytochemical compounds, including phenolic compounds (Figure 1). In general, fruits from the *Vaccinium* spp have positive effects on health linked to the bioactive properties of their phytochemical components [56, 57, 58, 59, 60]. Various studies have confirmed their anti-inflammatory and anticarcinogenic properties, as well as their cardiovascular protective effects. Moreover, they also present anti-neurodegenerative effects and have anti-microbial properties [59, 61].

The predominant bioactive compounds in blueberries are phenolic compounds, which represent up to around 0.3% of the total content of the fruit, as was assayed by Moyer *et al* using the Folin-Ciocalteu method to determine total soluble phenolics (TPH) [62]. This percentage varies according to factors related to the cultivation, growth, and maturity of the berry, as well as the analytical method used for its quantification [56]. According to various reports, the most abundant phenolic compounds in berries of the *Vaccinium* genus are procyanidins; phenolic acids; stilbene derivates such as resveratrol; and flavonoids, which mainly include flavonols and anthocyanins such as malvidin, cyanidin, delphinidin, petunidin, and peonidin [59, 63, 64, 65]. These bioactive compounds have different mechanisms of action, such as induction of metabolizing enzymes, regulation of gene expression, and modulation of various signaling routes [66]. Other phytochemicals present in blueberries are carotenoids [67, 68], phytosterols [69] and isoprenoids [70].

### 3.2 Antioxidant potential and antiproliferative capacity of *Vaccinium spp* berries in skin cancer cells*: in vitro and in vivo studies*

In some studies on skin cancer, mostly based on cell culture and animal models, diverse phenolic compounds were reported to be related to the induction of apoptosis, the inhibition of cellular proliferation, angiogenesis, and cell cycle arrest [71]. Moreover, the inhibition expression of inflammatory cytokines genes, including IL-6, IL-1, GM-CSF and TNF-α was identified in melanoma models [72]. Reduction expression in these genes is associated with the antioxidant effect of phenolic compounds since the inflammatory process induces oxidative stress and reduces cellular antioxidant capacity. In other studies was found that fisetin, which belongs to the flavonoid family and is found in fruits and vegetables including for instance mangos, strawberries, apples, kiwis, grapes and onions, has antioxidant properties and antiproliferative effects against various types of cancer [73]. In melanoma cells, 60 uM of fisetin effectively inhibits cell growth and its action is related to the interruption of the WNT/β- catenin signaling pathway [74]. Another study reported that topical application 250 and 500 nmol of fisetin to SKH-1 mice after ultraviolet radiation exposure, results in inhibited PI3K/AKT/NF-κB signaling, which is associated with UVB-induced inflammation, cell survival, and proliferation [75].

Specifically, the chemopreventive properties of *Vaccinium spp* berries have been studied and were established that they can act through different mechanisms to counter damage to cutaneous cells and prevent or delay the progression of skin cancer. Various *in vitro* and *in vivo* studies have demonstrated that these mechanisms of action include the activation of the antioxidant system and the inhibition of tumor cell proliferation, through the regulation of the cell cycle or the activation of apoptosis. The relevant findings are summarized in Table 1.

**Table 1:** Evidence of the chemopreventive effect of bioactive compounds of blueberries against skin cancer.

Bioactive products derived from blueberry	Doses	Model	Effects on skin cancer	References
Anthocyanin-rich extract	200y400μg/ml	Cell line B16–F10	Proliferation in B16–F10 cells was inhibited in a dose- dependent manner.	[61]
Resveratrol	$10^{-2},10^{-1},\;1.5,\;10\;\mu M$	Cell line A375SM	The viability of the cellular line of melanoma was significantly reduced in a dose-dependent manner.	[76, 77]
Resveratrol	1,10μM	Cell line A375SM	- Expression of p21 y p27 was activated, which induced cell cycle arrest in melanoma cells. - Apoptosis was induced.	[76, 77]
Delphinidin	10mM	Cell line HaCaT	Pre-treatment with delphinidin prevented apoptosis induced by UVB in HaCaT cells.	[78]
Delphinidin	1−20mM	Cell line HaCaT	Strong dose-dependent antioxidant activity and inhibition of lipid peroxidation induced by UVB rays was evidenced in HaCaT cells.	[78]
Agraz extract (*Vaccinium meridionale Swartz)*	10,50and100μg/ml	Cell line HT1080	Agraz extract provided significant protection in cell viability at the concentrations of 50 and 100 μg/ml for 48 h before exposure to rotenone-induced oxidative stress.	[79]
Cyanidin-3-O- (6-OP-coumaril) -monoglucoside + Na	$10\frac{mg}{ml}acai\;extract$	BALB/3T3 Fibroblasts	The production of ROS induced by UVA in immortalized fibroblasts was mitigated.	[80]
Malvidin-petunidin-glucoside-epicatechin	$10\frac{mg}{ml}acai\;extract$	BALB/3T3 Fibroblasts	The production of ROS induced by UVA in immortalized fibroblasts was mitigated.	[80]
Cyanidin - 3 - glucoside	30μg/ml	Primary keratinocytes C57BL/6	Reduced oxidation mediated by UV in keratinocytes.	[81]
Berry extract (wild blueberry, bilberry, cranberry, elderberry, raspberry seed and strawberry)	250μg/ml	Cell line HaCaT	Potential inhibition of the expression of $H_{2}O_{2}$ and the induction of VEGF induced by TNFα in human keratinocytes.	[82]
“Bluecrop” blueberry extract (BE)	$6\;\frac{mg}{ml},\;8\;\frac{mg}{ml},\;10\frac{mg}{ml}$	Cell line HaCaT, HFF	Treatment with BE reduced the levels of transcription of inflammatory cytokine genes including TNF-α, IL-1β, IL-8, and IL-6.	[83]
Delphinidin	$\;1\frac{mg}{100ml}\;DMSO/mouse$	SKH-1 Mice	Topical application of delphinidin in SKH-1 hairless mice inhibited apoptosis mediated by UV and DNA damage markers, such as cyclobutane pyrimidine dimers.	[78]
Cyanidin-3-glucoside	250y500μM	SKH-1 Mice	Inhibited DNA damage and skin inflammation in SKH-1 hairless mice.	[84]

## 4 Conclusions and perspectives

The evidence indicates that oxidative stress generated as a consequence of prolonged exposure to UV radiation has a role in the development of skin cancer. This has led to research on antioxidant therapy as a mechanism to prevent or delay the process of carcinogenesis. In this area, a focus for study are the compounds derived from natural products, and one of the sources evaluated are berries of *Vaccinium spp*. As reviewed, several studies evaluated bioactive compounds of these berries, both *in vitro* and *in vivo* models. Results showed protective effects on skin cells associated with blueberries phenolic compounds that included inhibition of proliferation and cell cycle arrest in malignant cells, decreased oxidized macromolecules, down-regulation of inflammatory cytokine genes, and mitigated oxidative stress. These biological responses suggest that a chemopreventive option against skin cancer could be the inclusion of these substances in the daily diet.

The findings summarized reflect advances in the study of the antioxidant potential and antiproliferative capacity against skin cancer of phytochemicals derived from blueberries. However, it remains necessary to elucidate the mechanisms that regulate these processes and to explore in more detail some species, such as the Colombian blueberry (*Vaccinium meridionale Swartz)* that in other studies was reported as a source of anthocyanins and phenolic acids.

## Declarations

### Author contribution statement

All authors listed have significantly contributed to the development and the writing of this article.

### Funding statement

This work was supported by the grant P20250 from Instituto Tecnológico Metropolitano.

### Data availability statement

No data was used for the research described in the article.

### Declaration of interests statement

The authors declare no conflict of interest.

### Additional information

No additional information is available for this paper.
